# Undernutrition and associated factors among pregnant women in Ethiopia. A systematic review and meta-analysis

**DOI:** 10.3389/fnut.2024.1347851

**Published:** 2024-05-06

**Authors:** Shimeles Biru Zewude, Mekonen Haile Beshah, Mengesha Assefa Ahunie, Dawit Tiruneh Arega, Dagne Addisu

**Affiliations:** ^1^Department of Midwifery, College of Medicine and Health Sciences, Debre Tabor University, Debre Tabor, Ethiopia; ^2^Department of Social and Public Health, College of Medicine and Health Sciences, Debre Tabor University, Debre Tabor, Ethiopia

**Keywords:** undernutrition, women, associated factors, meta-analysis, Ethiopia

## Abstract

**Background:**

Maternal undernutrition is a major public health concern due to its association with mortality and overall disease burden for mothers and their children. Maternal nutrition determines pregnancy outcomes since reduced intake of nutrients influences gestational age length, placental function, and fetal growth during pregnancy. The complexity of the intergenerational aspects of maternal nutrition may also confound the design of interventions. Therefore, this research aimed to assess the prevalence of undernutrition and associated factors among pregnant women in Ethiopia.

**Methods:**

We identified the literature from PubMed, EMBASE, Scopus, and CINAHL databases. Data were entered into Microsoft Excel and then exported to Stata version 17 statistical software for analysis. The I^2^ and Q-statistic values detect the level of heterogeneity, and meta regression was performed to investigate between-study heterogeneity using more than one moderator. JBI quality assessment tools were used to include relevant articles. Evidence of publication bias was indicated using the funnel plot and Egger's linear regression test. The effect size was expressed in the form of point estimates and an odds ratio of 95% CI in the fixed-effect model.

**Result:**

In total, 19 studies fulfill the inclusion criteria. The pooled prevalence of undernutrition among pregnant women was 32% (95% CI 31.3–33.2 I^2^ = 97.5%, P < 0.0). Illiteracy (AOR = 3.6 95% CI; 2.3–5.6), rural residence (AOR = 2.6 95% CI; 1.2–3.5), a lack of prenatal dietary advice (AOR = 2.6 95% CI; 1.8–3.7), household food insecurity (AOR = 2.5 95% CI; 1.9–3.2), and low dietary diversity score (AOR = 3.7 95% CI; 2.2–5.9) appear to be significantly associated with undernutrition among pregnant women.

**Conclusion:**

The review showed that the prevalence of undernutrition is still high among pregnant women. Illiteracy, rural residence, a lack of prenatal dietary advice, household food insecurity, and low dietary diversity score were significantly associated with undernutrition during pregnancy. Interventions should focus on educating the public and helping families access food or supplements they need through local markets, health systems, and community-based support, as undernutrition is caused by numerous interconnected causes.

**Systematic review registration:**

https://www.crd.york.ac.uk/prospero/#myprospero, identifier: CRD42023417028.

## Background

According to the World Health Organization (WHO), undernutrition is one of the top 10 ranked risk factors for the burden of disease worldwide (1). It results from either inadequate dietary intake in terms of quantity or quality, inadequate nutrient utilization brought on by infections or other disorders, or from either of the two (2). During the first 1,000 days of a child's life (from conception to 6 months of a baby's life), the mother is the only source of nutrition for the infant via the placenta and then through exclusive breastfeeding during the first 6 months of life (3).

Too many women, particularly young people and those who are nutritionally vulnerable, do not receive the nutrition they require for maintaining their health and providing their unborn children the greatest possible chance to live, grow, and develop (4).

Due to the physiological demands of pregnancy and lactation, pregnant women are frequently nutritionally susceptible (5). Inadequate dietary intakes before, during, and after pregnancy have the potential to harm mothers and their offspring. Comprehensive improvements in women's nutritional and physical wellbeing before and during pregnancy will support healthy fetal development, better labor and delivery outcomes, greater prenatal survival, and perhaps even longer-term health for both the mother and child. However, the quality of diet for pregnant women in many resource-poor settings is very low, and there are discrepancies between intakes and requirements for a variety of micronutrients (6, 7).

Approximately half of the deaths in children under 5 years in low- and middle-income countries are directly or indirectly caused by mothers who are undernourished (8). Based on a pooled estimate carried out in 137 developing nations, maternal undernutrition was responsible for 14.4% of stunting among 44.1 million children of < 2 years of age (9). On the other hand, mothers with better nutritional status take better care of themselves and provide their children with better care (10). Because an undernourished mother is more likely to give birth to an undernourished child, the cycle of undernutrition is perpetuated through generations (11–13).

Undernutrition during pregnancy can lead to preterm birth, intrauterine growth restriction, and reproductive loss through stillbirths. Recent research suggests that healthy eating throughout pregnancy and breastfeeding may also lower the child's risk of developing chronic diseases later in life (14–16). A vulnerability to undernutrition *in utero* is associated with stunted growth and development in children, short stature in adults, lower intellectual attainment, and lower economic production (17, 18).

Maternal undernutrition is commonly cited as a cause of retarded fetal growth, malformations associated with the central nervous system such as spinal bifida and hydrocephalus and prematurity (19, 20). Serious issues of maternal undernutrition are evident in most countries in Sub-Saharan Africa and south-central and southeastern Asia (7, 21). Previous studies have established that undernourished pregnant women suffer from a combination of chronic energy deficiency that leads their children to have a low birth weight (LBW) and also causes preterm birth and unsuccessful birth outcomes (22–24). Regardless of significant gains and signs of progress in the last decade, maternal undernutrition still remains a major public health problem in Ethiopia. Because the causes of malnutrition are complex, they should be addressed in a systematic way to find the right solutions for the problem. In general, malnutrition is not the result of a single consequence of a single factor but is caused by a mixture of different factors, and the extent of the contribution of each factor may vary. Therefore, this systematic review and meta-analysis aimed to assess the magnitude of undernutrition and associated factors among pregnant women in Ethiopia.

## Methods

### The protocol and registration

This systematic review and meta-analysis were reported using the Preferred Reporting Items for Systematic Review and Meta-Analysis Protocols (PRISMA-P) (25) (Supplementary File 1). The protocol for this review was registered on the (PROSPERO) International Prospective Register of Systematic Reviews (registration number 2023: CRD42023417028).

### Search strategy

We identified literature from PubMed, Medline, EMBASE, Scopus, and CINAHL databases (Supplementary File 2). A supplementary search was conducted to find gray literature from the Google search engine and Google Scholar. In addition, we contacted three authors to request for additional information missing from their papers. The study includes articles published from January 2016 to March 2023 in Ethiopia and published in English. We employed the Medical Subject Headings (MeSH) terms and combined keywords to identify studies in these databases.

### Eligibility criteria

The study includes case-control, cross-sectional, and cohort studies, irrespective of sample size, published from January 2016 to March 2023 and published only in English. The review included articles defining undernutrition during pregnancy according to the researchers' categories and studies. We excluded the studies if they were not related to the title, were duplicates, were not carried out in Ethiopia, were anonymous reports, and were not published in English. The primary outcome of interest in this study is the prevalence of undernutrition among pregnant women.

### Study selection procedure

We identified an initial set of studies by using the search terms, and then the retrieved studies were exported to the reference manager software, EndNote version 7. Duplicated studies were removed using the EndNote and also removed manually. Two independent reviewers (SB) and (MH) screened the title and abstract for relevance. During this preliminary assessment, primary studies that were found to be irrelevant were excluded. Differences between the two reviewers were resolved by consensus, or if necessary, the third reviewer (MA) was consulted for a disagreement based on the relevance of the stated objectives and inclusion criteria. Finally, studies with relevant information and those that fulfill inclusion criteria were selected for full-text review, and excluded studies were reasoned out via a flow chart.

### Data collection process

We used an Excel spreadsheet for data extraction. Two reviewers (DA and DT) extracted the data using a data extraction format that includes authors, year of publication, study area, study design, and sample size. We extracted data on the percentage and factors affecting undernutrition among pregnant women with adjusted odd ratios. The extracted data were then edited and saved in a comma-delimited file for analysis.

### Outcomes of interest

The study has two main outcomes: the prevalence of undernutrition among pregnant women and the factors associated with maternal undernutrition during pregnancy. The odds ratio was calculated for the common risk factors of the reported studies.

### Individual study's risk of bias

The authors rigorously assessed the quality of all the selected studies for inclusion in the review. The JBI quality assessment tool was used to assess the quality of each individual study (26) (Supplementary File 3). For each study design, we included a study that scored above the mean value of the checking points.

### Data synthesis and analysis

The data extracted and saved in the comma-delimited file format in an Excel spreadsheet were imported to Stata version 17.0 (Stata Corporation, College Station, TX, USA) statistical software for analysis. We used forest plots to graphically report the results. We checked the presence of heterogeneity among studies using the chi-squared test where a *p* < 0.05 denoted statistical significance. The I-squared (I^2^) statistic was used to quantify the level of heterogeneity among the studies. We assumed substantial heterogeneity among studies when the value of I^2^ was ≥50%. We used the fixed-effects model to conduct meta-analyses where the studies did not have substantial heterogeneity (i.e., I^2^ statistic < 50%). If the I^2^ statistic is ≥50% (as suggested by Higgins et al.), indicating high heterogeneity (27), a random effect model was used. We pooled the percentages of undernutrition during pregnancy. We carried out subgroup analyses according to different categories of publication years and regions, and meta regression was performed to investigate between-study heterogeneity by using more than one moderator by the random effect model (Sidik–Jonkman method) instead of the default restricted maximum-likelihood (REML) method. Sensitivity analyses were conducted using a leave-one-out meta-analysis to explore the influence of a single study on the overall effect size estimate on maternal undernutrition during pregnancy. Publication bias was assessed by an inspection of the funnel plot and formal testing for asymmetry of the funnel plot using Egger's linear regression test.

## Result

### Selection of studies

**Figure 1:**
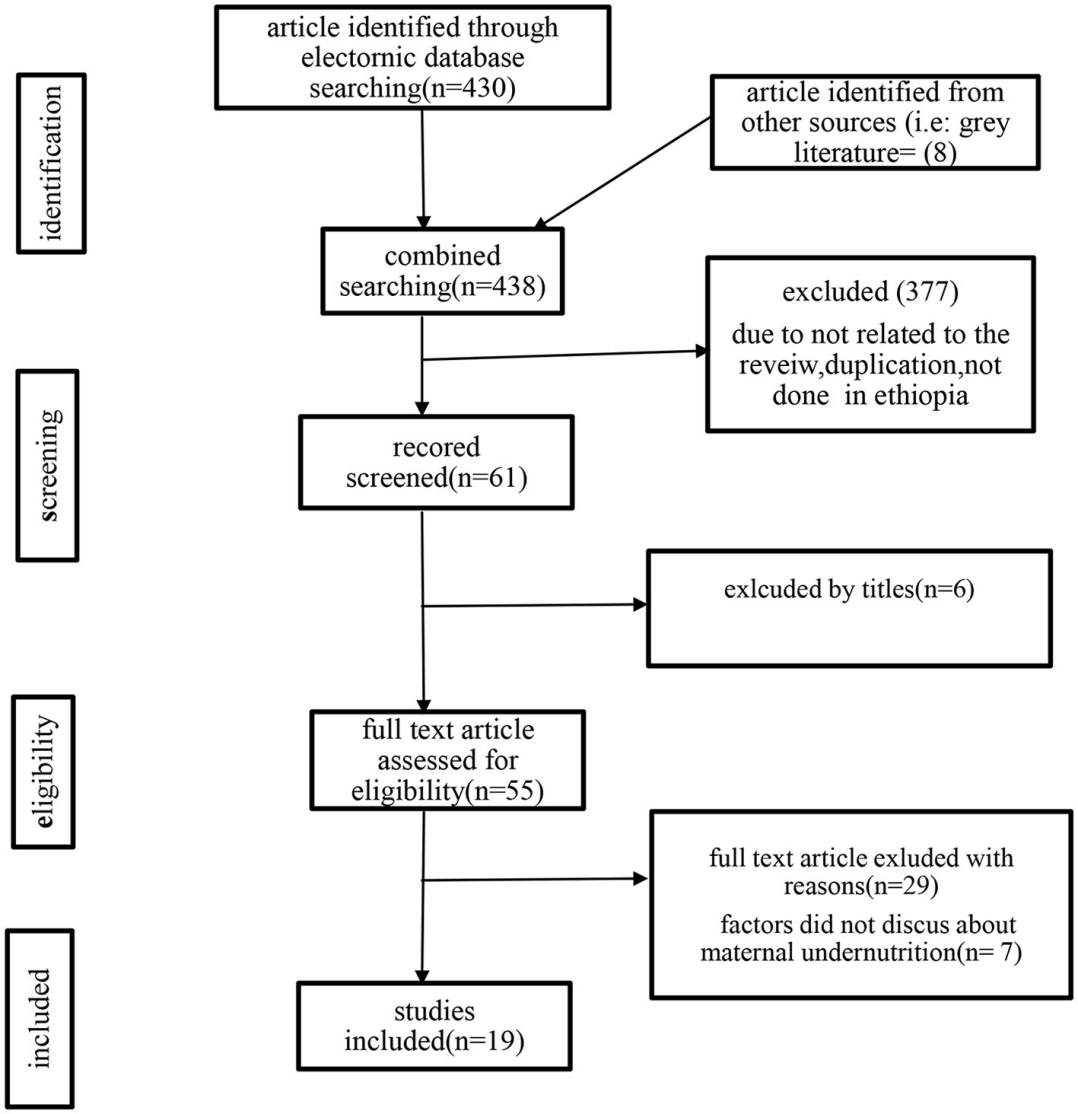
PRISMA flow diagram of search results for included studies.

The database search yielded 430 studies; similarly, the gray literature yielded eight articles. In total, the search retrieved 438 articles. A total of 343 studies were not related to our title and not carried out in Ethiopia, 34 articles were found to be duplicates, and six articles were excluded since they were not related to our titles and were confirmed irrelevant to this review. Finally, 55 potential full-text articles were screened. From full-text articles, 36 were excluded for the following reasons: 29 articles had wrong outcomes and seven studies did not discuss factors affecting maternal undernutrition. Finally, 19 studies were included in this review (Figure 1).

### Study characteristics

Regarding the study area, seven of the studies were conducted in the Oromia region (23, 28–33), six studies in the South Nation Nationalities and People region (SNNPR) (34–39) two studies each in the regions of Amhara (22, 40) and Tigray (41, 42), and one in the Afar (43) and Gambella regions (44) (Table 1). The most commonly associated factors included in this review were illiteracy, rural residence, pregnant women who were married before the age of 18, no antenatal care follow-up, substance use during pregnancy, a lack of prenatal dietary advice, not participating in women's Health Development Army meetings, intestinal parasite infection, skipping meals, household food insecurity, and low dietary diversity.

**Table 1:** The characteristics of studies included show undernutrition among pregnant women in Ethiopia.

**References**	**Region**	**Study design**	**Study period**	**Sample size**	**Operational definition (cm)**	**Prevalence (%)**
Birara Aychiluhm et al. (43)	Afar	Facility-based cross-sectional study	From 20 September to 5 October 2020	387	MUAC < 22	30.9
Endalifer et al. (41)	Tigray	Facility-based cross-sectional study	From 1 April to 20 June 2017	321	Below 22.5	22.3
Fite et al. (32)	Oromia	Community-based study	From 10 March to 31 March 2015	434	MUAC < 22	34
Arero (29)	Oromia	Community-based study	From November 2020 to December 2021	420	MUAC < 23	44.9
Gebremichael et al. (30)	Oromia	Community-based study	From 30 January to 30 April 2021	1026	MUAC < 23	43.8
Nigatu et al. (44)	Gambella	Community-based study	From March to April 2014	338	MUAC < 21	28.6
Tesfaye et al. (34)	SNNPR	Facility-based cross-sectional study	From 01 Feb to 01 March 2019	242	MUAC < 22	21
Dadi et al. (40)	Amhara	Community-based study	From 15 June to 30 July 2018	940	MUAC < 22	14.4
Kumera et al. (22)	Amhara	Facility-based cross-sectional study	January and February 2016	409	MUAC < 22	16.2
Zewdie et al. (31)	Oromia	Community-based study	From 15 June to 30 June 2020	383	MUAC < 23	41.2
Fite et al. (32)	Oromia	Community-based study	From 5 January to 12 February 2021	475	MUAC < 23	47.9
Shiferaw et al. (23)	Oromia	Community-based study	From 30 June to 30 July 2018	382	MUAC < 23	44.9
Teshome et al. (35)	SNNPR	Facility-based cross-sectional study	From February to May 2019	344	MUAC < 23	52.6
Tilahun et al. (36)	SNNPR	Facility-based cross-sectional study	March–May 2021	576	MUAC < 23	42.4
Gelebo et al. (37)	SNNPR	Community-based study	From December 2018 to January 2019	527	MUAC < 23	43.1
Tafara et al. (33)	Oromia	Facility-based cross-sectional study	From 10 April to 10 May 2020	806	MUAC < 23	39.2
Muze et al. (39)	SNNPR	Facility-based cross-sectional study	From July to January 2019	422	MUAC < 23	21.8
Ayele et al. (42)	Tigray	Facility-based cross-sectional study	From 01 August to 30 December 2018	844	MUAC < 23	40.6
Gizahewu et al. (38)	SNNPR	Facility-based cross-sectional study	From 01 March to 30 April 2015	211	MUAC < 22	24.6

### Individual study's risk of bias

The Joanna Briggs Institute (JBI) quality appraisal checklist was used for each study design. The disagreement between two reviewers (SB and MH) was resolved by consulting a third reviewer (MA). Cohen's Kappa statistics was used to calculate the degree of agreement between the two reviewers (SB and MH). The inter-rater agreement between the authors for study inclusion, data extraction, and methodological quality was assessed using Cohen's kappa coefficient (values ≤ 0 indicates no agreement, 0.01–0.2 indicates none to slight, 0.21–0.4 indicates fair, 0.41– 0.6 indicates moderate, 0.61–0.8 indicates substantial, and 0.81–1 indicates almost perfect agreement). Kappa coefficient of >0.63 was accepted (45).

### Pooled estimate of undernutrition among pregnant women

**Figure 2:**
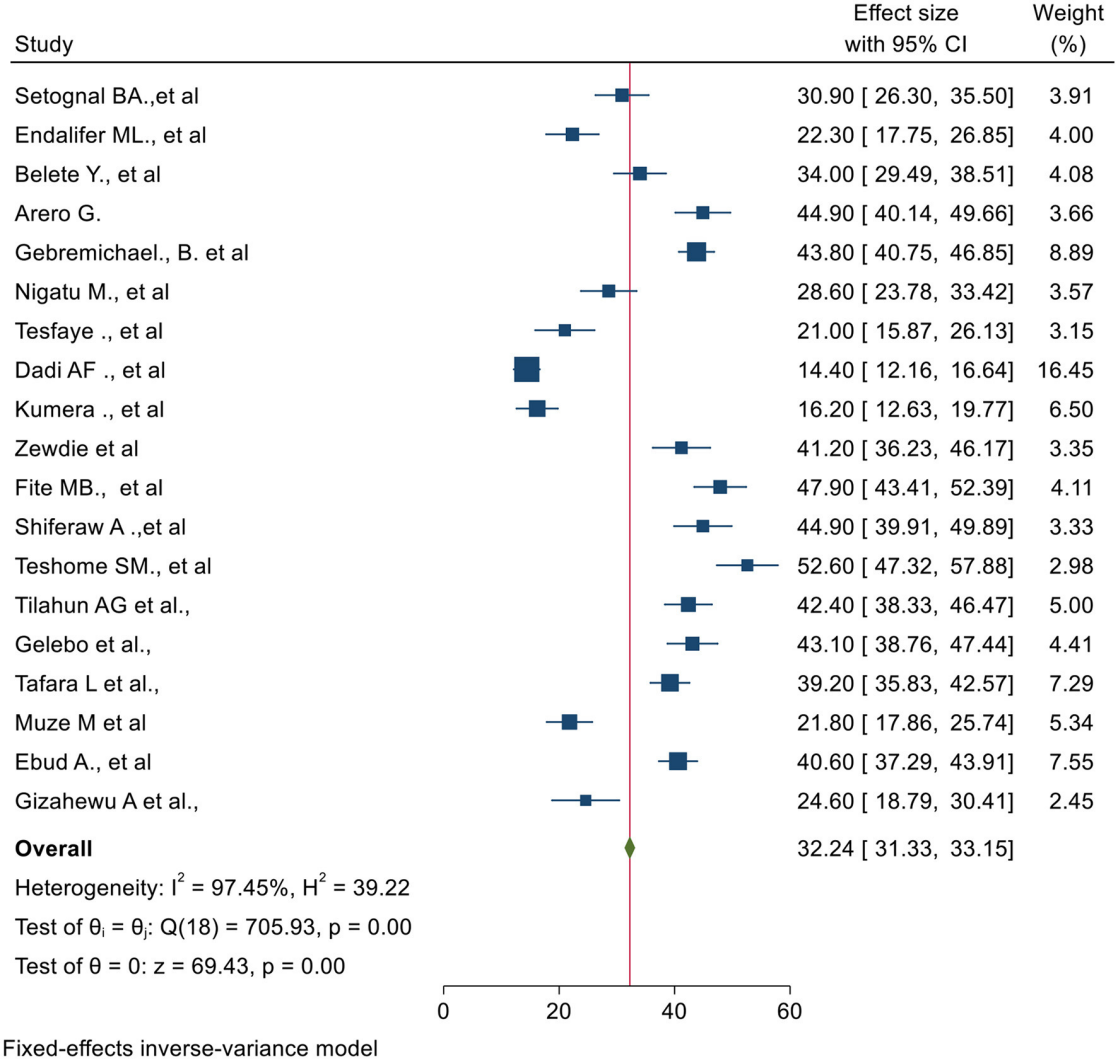
Forest plot for magnitude of undernutrition among pregnant women in Ethiopia, 2023.

The overall pooled prevalence of undernutrition among pregnant women is presented with a forest plot (Figure 2). Therefore, the estimated prevalence of undernutrition among pregnant women in Ethiopia was 32% (95% CI: 31.3–33.2 I^2^ = 97.5%, P < 0.0).

### Publication bias

**Figure 3:**
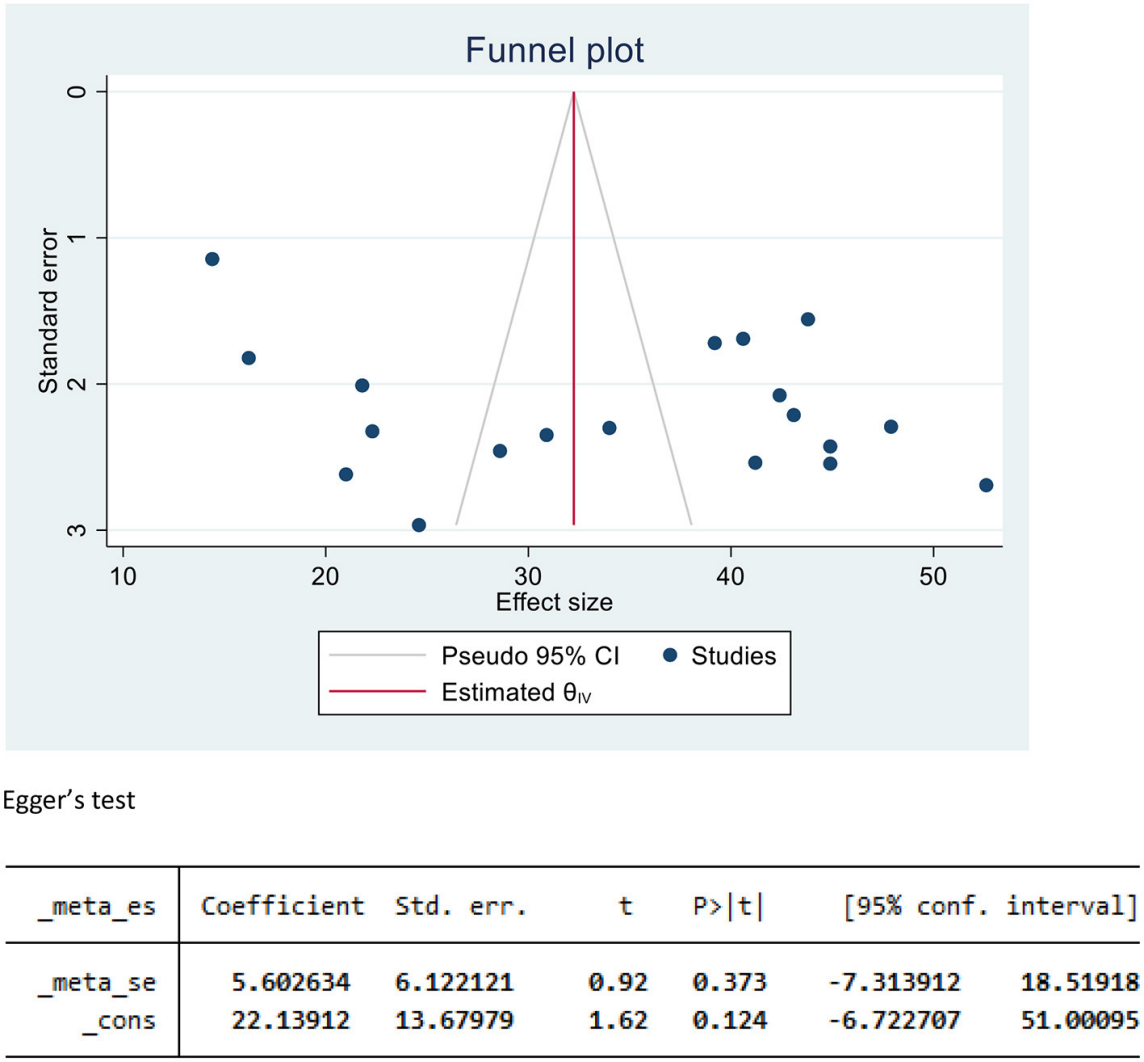
Funnel plot and Egger's test to assess publication bias for undernutrition among pregnant women in Ethiopia, 2023.

The presence of a small study effect was checked by using funnel plots and Egger's test from the log-odds ratio of the proportion for a better property of meta-analysis. The SE is dependent on the value of log odds and the underlying proportion. Hence, the funnel plot showed a symmetric distribution, and Egger's test value was 0.92; both results revealed that there was no publication bias (Figure 3).

### Subgroup analysis

**Figure 4:**
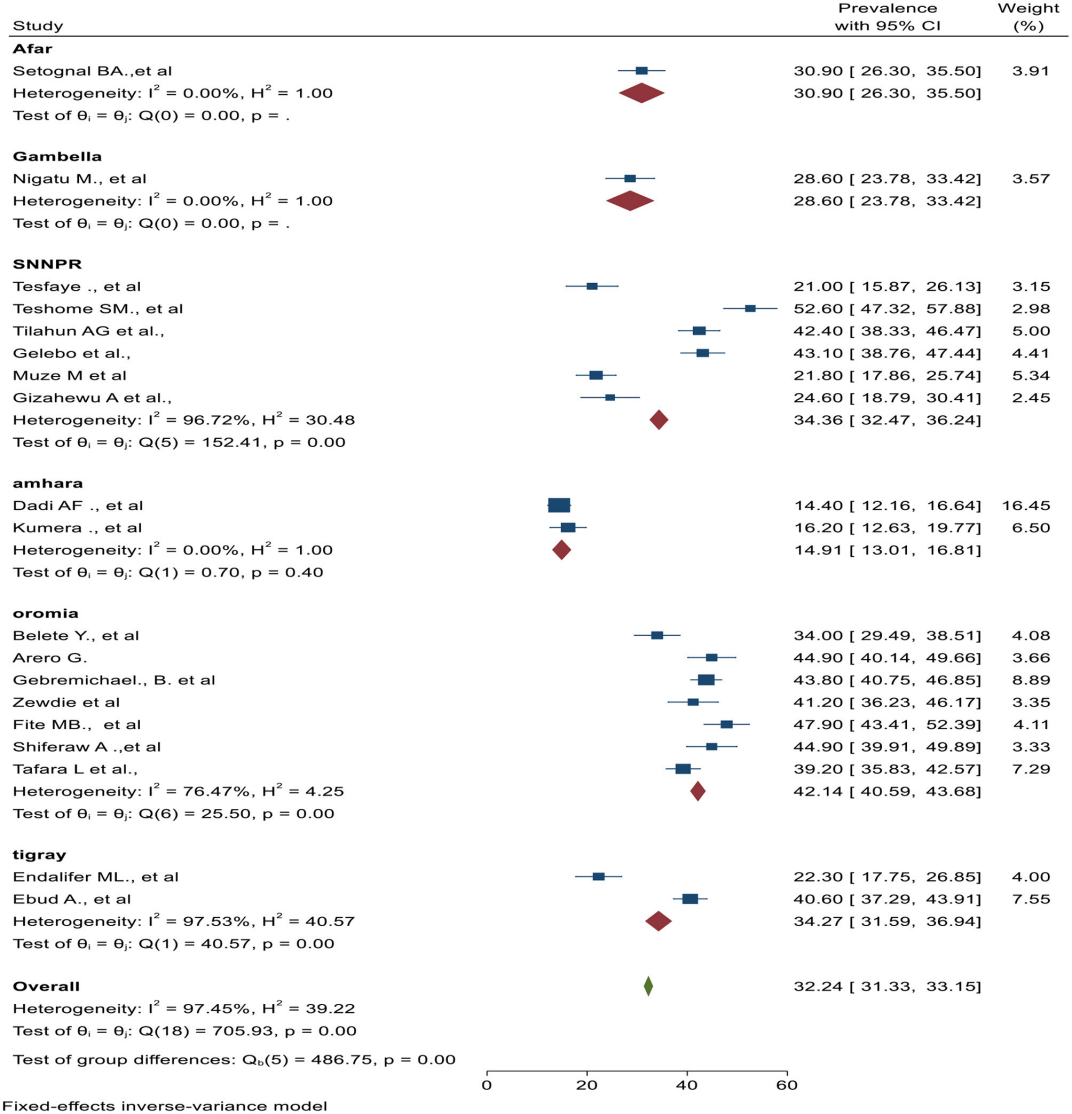
Subgroup analysis by region of studies and pooled effect of undernutrition among pregnant women in Ethiopia, 2023.

**Figure 5:**
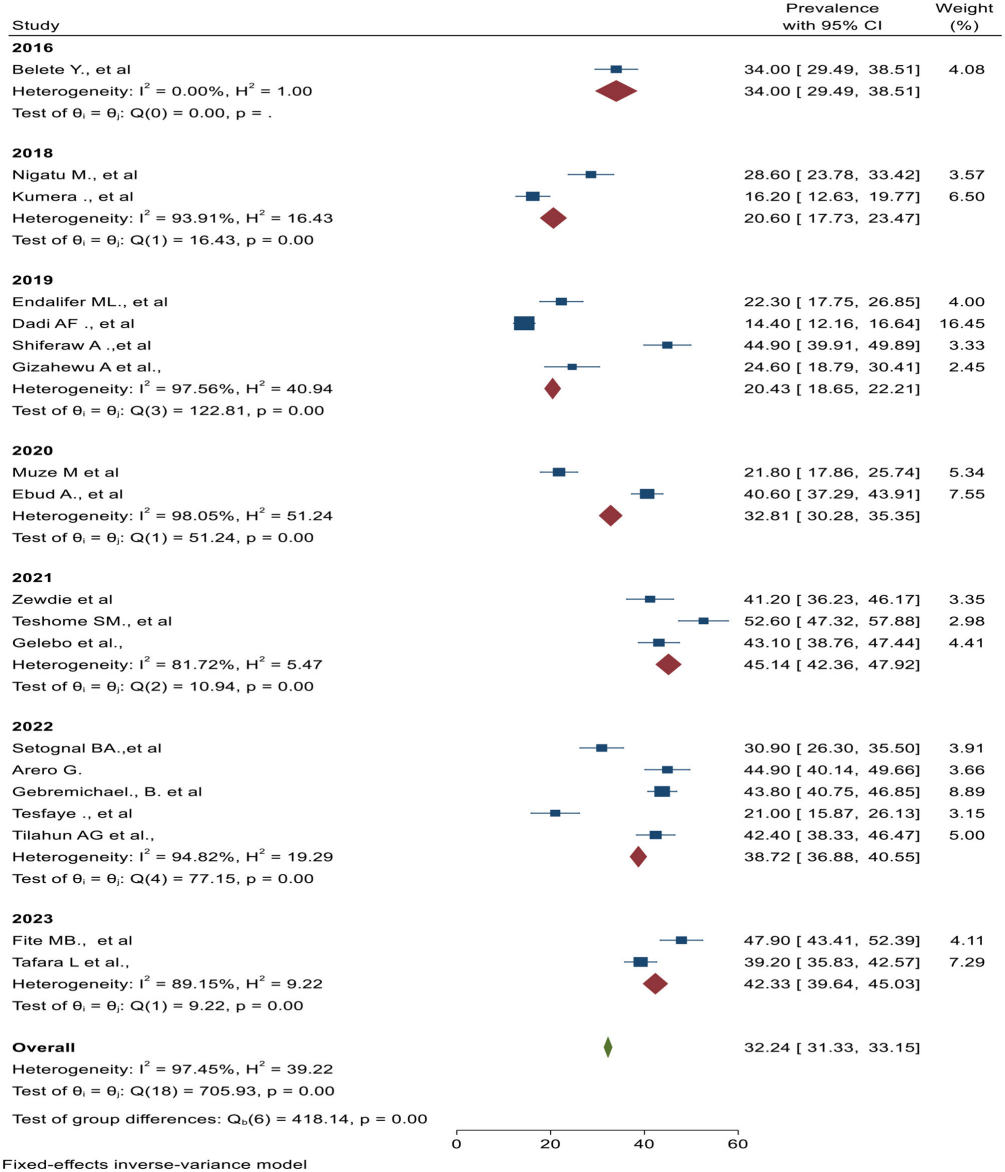
Subgroup analysis by publication year of studies and pooled effect of undernutrition among pregnant women in Ethiopia, 2023.

Subgroup analysis was employed with the evidence of heterogeneity using I-squared (I^2^) tests. In this study, the I^2^ statistic was 97.5%, *P* < 0.0, which showed evidence of marked heterogeneity. Therefore, subgroup analysis was carried out by using region and publication year as moderators (Figures 4, 5).

### Heterogeneity test using meta-regression

**Figure 6:**
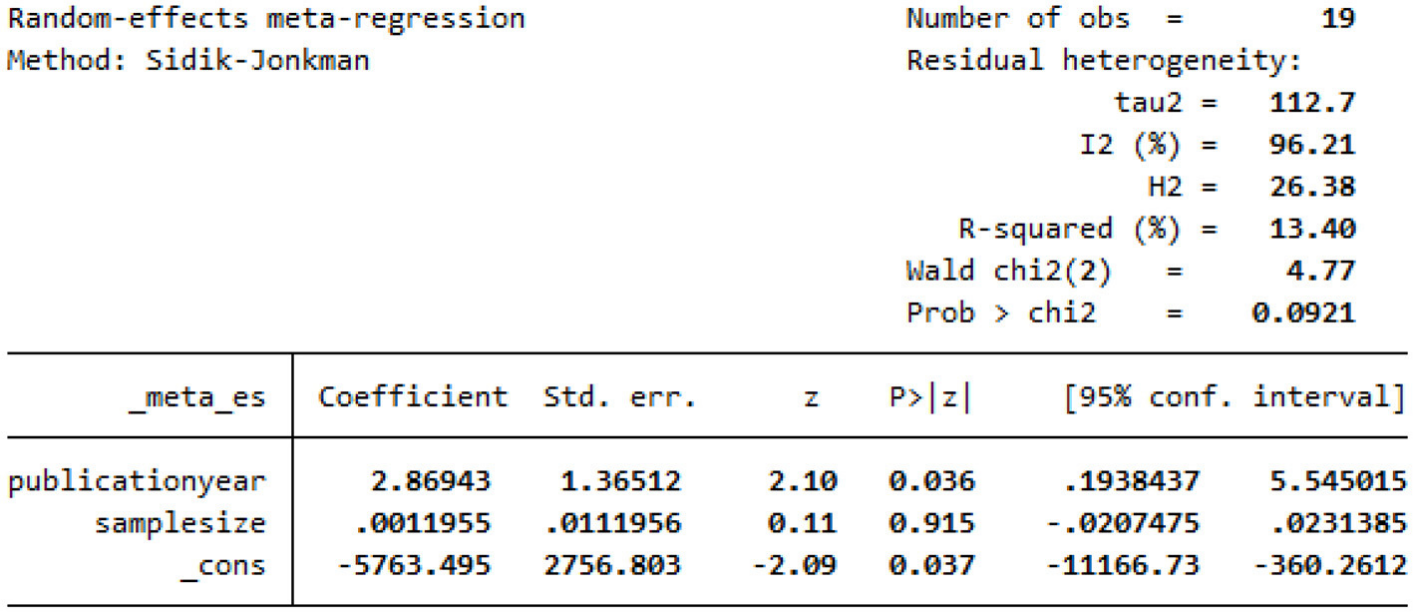
Meta-regression to assess heterogeneity and meta-analysis model using a publication year and sample size as moderators for undernutrition among pregnant women in Ethiopia, 2023.

Using meta-analysis regression, we collected a summary report of the heterogeneity statistics test among 19 primary studies included in this review. We used a different type of random effect model (Sidik–Jonkman method) instead of the default restricted maximum-likelihood (REML) method since REML assumes that the error distribution is normal, whereas the Sidik–Jonkman estimator does not. After the heterogeneity test, using meta regression reported an I^2^ statistic of 96%, which suggested very high heterogeneity even after including publication year and sample size as the moderators. The adjusted R^2^ statistic was used to assess the proportion of between-study variance explained by the covariates (publication year and sample size). In this study, approximately 14% of the between-study variance is explained by the covariate (Figure 6).

### Sensitivity analysis

**Figure 7:**
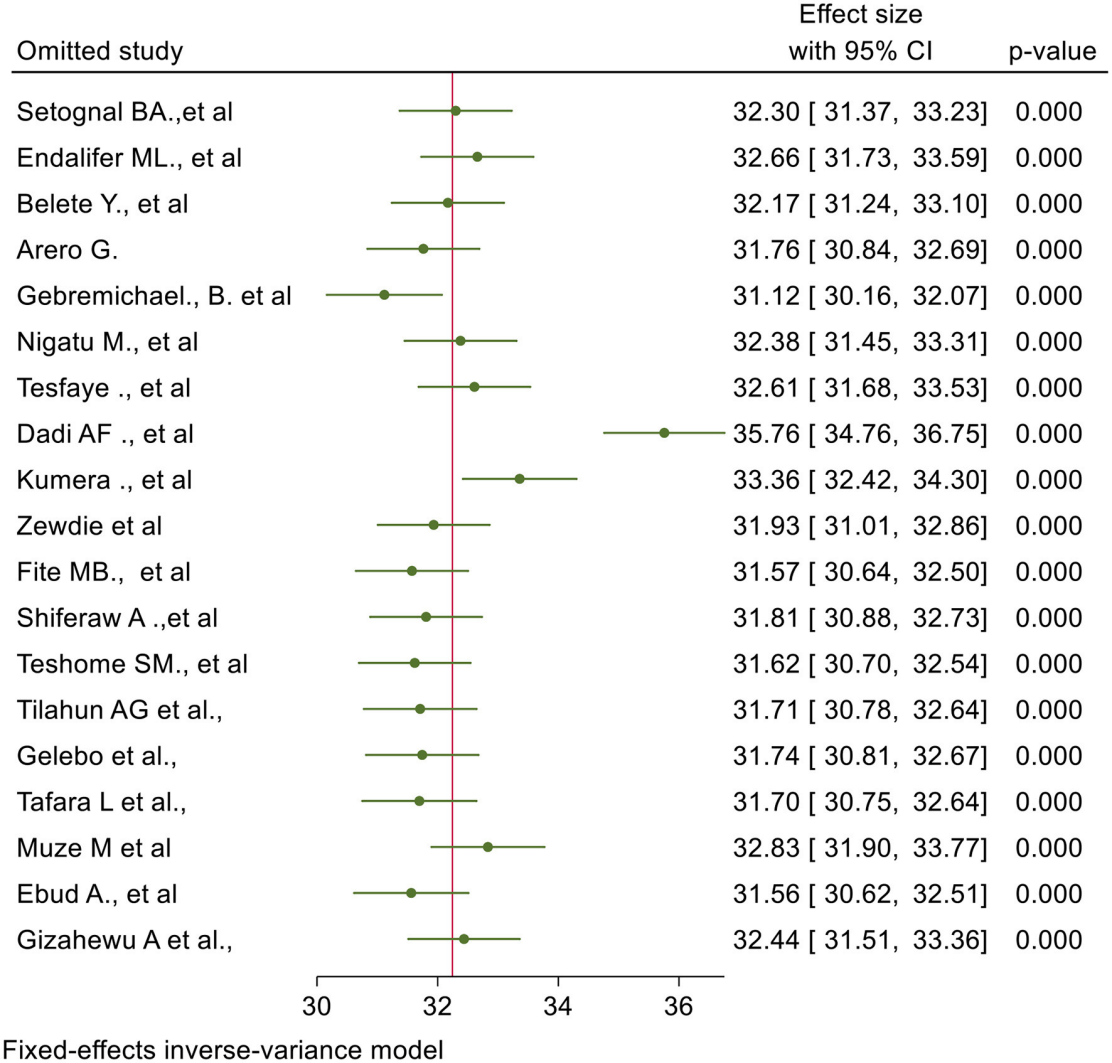
Leave one out meta analysis for studies included in a systematic review and meta-analysis of undernutrition during pregnancy in Ethiopia, 2023.

Leave-one-out meta-analysis was performed to explore the influence of a single study on the overall effect size estimate on maternal undernutrition during pregnancy. The prevalence of undernutrition among pregnant women was high (35.8%) in Dadi AF. et al.'s study and low (31.1%) in Gebremichael B. et al.'s study (Figure 7).

### Factors associated with undernutrition among pregnant women

Factors such as illiteracy, rural residence, pregnant women who were married before the age of 18, no antenatal care follow-up, substance use during pregnancy, a lack of prenatal dietary advice, not participating in women's Health Development Army meetings, intestinal parasite infection, skipping meals, household food insecurity, and low dietary diversity were associated with undernutrition among pregnant women.

Illiterate (AOR = 3.6 95% CI; 2.3–5.6) women were 3.6 times more undernourished than literate women during pregnancy. The odds of undernutrition among rurally residing (AOR = 2.6 95% CI; 1.2–3.5) pregnant women were 2.6 times more likely than their urban counterparts. The pregnant women who have had low prenatal dietary advice (AOR = 2.6 95% CI; 1.8–3.7) were 2.6 times more likely undernourished than women who got the prenatal dietary advice. Women who have had high household food insecurity (AOR = 2.5 95%CI; 1.9–3.2) had 2.5 times higher odds of developing undernutrition during pregnancy. The odds of undernutrition among pregnant women who have had low dietary diversity (AOR = 3.695%CI; 2.2–5.9) were 3.6 times more likely than their counterparts.

## Discussion

Undernutrition is defined as a person's inability to consume enough calories and other nutrients to meet their needs and maintain their health (46). Worldwide, nearly about 462 million pregnant women are malnourished, and Africa is the most heavily overburdened continent (14, 47). Undernutrition during pregnancy occurs due to a dual load caused by increased demands during pregnancy and inadequate intake of food (48). Undernutrition is a key contributor to maternal mortality and morbidity and adverse birth outcomes (49). This review aimed to identify the national burden of undernutrition among pregnant women and the factors associated with it.

The pooled estimate of undernutrition among pregnant women remains high in Ethiopia with a 34% prevalence 32% (95% CI 31.3–33.2). This is quite more prevalent compared with the neighboring country Sudan (12.5%) (50). However, this finding is in line with the World Health Organization (51) report on Africa with prevalence rates of undernutrition being above 15% (51). A review performed on Sub-Saharan Africa indicates that maternal undernutrition during pregnancy is 23.5%; however, heterogeneity of studies in the measurement of undernutrition during pregnancy affected the outcomes of interest (52). The finding provides remarkable evidence that in Ethiopia, undernutrition among pregnant women continues to be a public health problem, and its adverse birth outcomes are still not halted (34).

This review aims to identify illiterate pregnant women who are known to be at risk for undernutrition. Education at higher levels increases knowledge of healthy prenatal and nutritional practices. Illiteracy has a direct correlation to women's ignorance of the importance of eating a balanced diet while being pregnant (53). Although significant nutrition interventions have been made, nutrition remains the primary priority area in Ethiopia (54). Given this, evidence from the region and other comparable settings demonstrates that significant rates of undernutrition persist, even in areas and households where food is abundant. In order to properly plan nutrition interventions for vulnerable population groups including nursing mothers and pregnant women, nutrition knowledge is a crucial component of dietary practices (55, 56). Additionally, women with low levels of education may have little to no influence over financial decisions, consumables food purchases, and food distribution in the home, which may contribute to their poor nutritional health (22).

According to this review, women who lived in rural areas had 2.6 times the likelihood of being undernourished compared to women who lived in urban areas. This result is in line with a systematic review and meta-analysis that highlights the burden of malnutrition among pregnant women in Africa (52). This could be explained by the fact that women in rural areas might not have access to antenatal health care services, information about eating a balanced diet, and other maternal health services (57).

Our analysis found a link between maternal undernutrition and a lack of prenatal dietary advice. Mothers who did not receive prenatal dietary recommendations were 2.6 times more undernourished than mothers who did receive recommendations. These findings highlight the value of nutritional counseling and appropriate feeding practices as interventions. According to a study from Bangladesh, pregnant women who are counseled to alter their eating habits can satisfy the increased nutritional requirements (58). This finding explains the critical necessity for healthcare providers to set up suitable monitoring and feedback mechanisms for antenatal nutrition education and counseling and doing so could increase women's understanding of proper nutrition.

The analysis also found that one of the characteristics that were independently linked to undernutrition during pregnancy was a lack of maintaining household food security. Compared to pregnant women from food-secure homes, those from food-insecure households had a 2.5 times higher risk of being undernourished. When families lack access to food, they frequently fall short of their daily nutritional needs and have inadequate dietary intake, which causes women to be undernourished (59). Our finding of an increased prevalence of maternal undernutrition in food-insecure households may reflect inequitable intra-household food allocation, whereby the nutritional needs of the child or other household members are given priority over those of the mother (60–62).

Women with low dietary diversity scores were 3.6 times more likely to be undernourished than women with dietary diversity scores above the mean values. Scientific literature backs up the strong link between nutrient adequacy and dietary variety (63, 64). This has been explained by the fact that there is no single food that contains all the required nutrients for optimal health (65). In the majority of developing countries, micronutrient inadequacy continues to pose a substantial threat to public health (66). Unfortunately, pregnant women and other women of reproductive age are the most vulnerable due to their higher dietary needs (67). Iron deficiency-related anemia and other micronutrient deficiencies are still widespread in impoverished countries because pregnant women are not getting adequate nutrition (68–70).

### Limitations of the study

The search strategy was limited to studies published in English and observational studies, causing reporting bias. The estimated magnitude of undernutrition during pregnancy in Ethiopia may need precaution in the interpretation of findings because no data were found in some regions.

## Conclusion

This review showed that the prevalence of undernutrition is still high among pregnant women. Illiteracy, rural residence, a lack of prenatal dietary advice, household food insecurity, and low dietary diversity were significantly associated with undernutrition during pregnancy. Interventions should focus on educating the public and assisting families in obtaining the food or supplements they require through local markets, healthcare systems, and community-based assistance. It also emphasizes the need for better prenatal counseling on dietary diversity. The study also highlights that there is a nutritional disparity between pregnant women in urban and rural areas and that it is important to focus on the nutritional practices of rural women. Even though anemia and malaria may not be connected directly to nutritional habits, it is almost inevitable that malaria infection will be associated with anemia; thus, the interaction between these interconnected health determinants should be the prime agenda, especially in malaria-affected heavily endemic areas.

## Data availability statement

The original contributions presented in the study are included in the article/Supplementary material, further inquiries can be directed to the corresponding author.

## Author contributions

SZ: Conceptualization, Data curation, Investigation, Supervision, Writing—original draft, Writing—review & editing. MB: Formal analysis, Investigation, Methodology, Software, Supervision, Visualization, Writing—review & editing. MA: Data curation, Investigation, Software, Visualization, Writing—review & editing. DTA: Resources, Software, Supervision, Validation, Visualization, Writing—review & editing. DA: Conceptualization, Investigation, Validation, Visualization, Writing—original draft.
