# Supplementary material for: Undernutrition and associated factors among pregnant women in Ethiopia. A systematic review and meta-analysis
Source: Front Nutr. 2024 May 6;11:1347851. doi: 10.3389/fnut.2024.1347851 (PMC11103006; doi:10.3389/fnut.2024.1347851)
Supplement: Supplementary file 3 [file Table_3.docx]

**Additional file 3:** JBI quality assessment score used to assess included article for undernutrition and associated factors among pregnant women in Ethiopia. A systematic review and Meta analysis.

| **Study ID** | **Author (year)** | **Weaknesses** | **JBI score** | **Quality status** |
| --- | --- | --- | --- | --- |
| 1 | Setognal et al 2022 | Potential confounder was explored like maternal age, partner education, family size, no of ANC visit.  Tool adapted national nutrition programme guideline and the Ethiopian demographic and health survey(CSA).  Effect of each independent variable on the other independent variable is not well stated. Detection bias more likely. | 82% | Low risk |
| 2 | Endalifer et al 2019 | Data were collected using a structured questionnaire. The outcome measurement was unlikely to be influenced.  The study considered small sample size.  No information is reported about missing data.  No causal relationship between maternal nutrition and its predictors. | 65.5% | Low risk |
| 3 | Belete et al 2016 | Adolescent pregnant women between the ages of 10-19 years in the randomly selected kebeles(lowest administrative unit) of the district were the study population. Selection bias is high likely.  multivariate logistic regression performed to control for all possible confounders | 75% | Low risk |
| 4 | Arero . 2022 | Factors were not exhaustively addressed.  The independent variables affect each other but it fails to show modality to express their level of correlation and measure taken to reduce its influence. So detection bias high likely. | 65% | Low risk |
| 5 | Nigatu et al 2018 | Data were collected using a structured questionnaire.  However there is no information about blinding of data collector. Authors not clearly state the outcome variables. | 70% | Low risk |
| 6 | Gebremichael. et al 2022 | Open Data Kit (ODK) applied to gather and record the data and WHO Self-Reporting Questionnaire was used so data collector manipulation of data was unlikely.  However tool validity in the study setting was not performed. | 85% | Low risk |
| 7 | Tesfaye et al 2022 | Structured validated tool was used. Maternal nutritional status assessed using a Mid Upper Arm Circumference (MUAC) measurement.  No causal relationship | 80% | Low risk |
| 8 | Dadi et al 2019 | Structured validated tools were used.  Maternal nutrition was assessed using a Mid Upper Arm Circumference (MUAC) measurement bias is unlikely. | 85% | Low risk |
| 9 | Kumera et al 2018 | A structured and pre-tested questionnaire was used, MUAC measurement, hemoglobin and stool specimen examination performed outcome measure unlikely to be manipulated.  Explored specific factors that affect maternal undernutrition among pregnant women. | 80% | Low risk |
| 10 | Zewdie et al 2021 | All pregnant women in any trimester residing in the district were the source population so all eligible participants were recruited.  Not establish a temporal relationship between undernutrition and its predictors. | 75% | Low risk |
| 11 | Fite et al 2023 | Measurement of outcomes was performed by using against the standard.  The potential confounding variables of maternal undernutrition was controlled.  Not establish a temporal relationship between undernutrition and its predictors. | 85% | Low risk |
| 12 | Shiferaw et al 2019 | All pregnant women in any trimester residing in the district were the source population so selection bias less likely.  The independent variables affect each other but it fails to show modality to express their level of correlation and measure taken to reduce its influence. So detection bias high likely. | 62% | Low risk |
| 13 | Teshome et al 2021 | used standardized tool for measurement of wealth index, nutritional status adopted from EDHS and USAID’s Food and Nutrition  Technical Assistance (FANTA-2007).  Explored specific factors that affect maternal undernutrition among pregnant women.  No causal relationship between maternal nutrition and its predictors. | 66% | Low risk |
| 14 | Tilahun et al 2022 | Validity of the questionnaires was checked by relevant professionals against the conceptual framework of the study and its reliability was checked by using a test-retest method.  Confounding variables controlled by multivariate logistic regressions. Multicollinearity is not well diagnosed. | 80% | Low risk |
| 15 | Gelebo et al 2021 | Study variable already stated and potential confounder considered like household income source of water, history of stillbirth.  All pregnant women who were residents of the Konso district for at least 6 months or above were included in the study so selection bias less likely.  Tool validity and reliability was done but its result was not properly explained and its adaptability in the study setting is not expressed well. | 83% | Low risk |
| 16 | Tafara et al 2023 | Pretested, structured tools were used. Maternal nutrition was assessed using a Mid Upper Arm Circumference (MUAC) measurement bias is unlikely.  Study participant recruited after Third-trimester pregnancy comes for antenatal follow up so it may not include factors occurred at early time of pregnancy. | 75% | Low risk |
| 17 | Muze et al 2020 | Adopt national ( EDHS,2016 ) and international(FAO knowledge, attitude, and practice survey guideline) standardized data collection tools. Outcome measurement influence less likely.  It is institution based study so it luck general conclusion of population. | 80% | Low risk |
| 18 | Ebud., et al 2020 | Third-trimester pregnancy women who were coming for delivery and antenatal care visits in general public hospitals of the Tigray region were selected.  Study participant recruited after Third-trimester pregnancy who comes for antenatal follow up so section bias is likely. | 75% | Low risk |
| 19 | Gizahewu et al 2019 | Pretested, structured tools were used. Maternal nutrition was assessed using a Mid Upper Arm Circumference (MUAC) measurement bias is unlikely.  No causal relationship between maternal nutrition and its predictors.  It is institution based study so it luck general conclusion of population. | 73% | Low risk |
